# Executive functioning in Cornelia de Lange syndrome: domain asynchrony and age-related performance

**DOI:** 10.1186/s11689-017-9208-7

**Published:** 2017-08-15

**Authors:** Donna Reid, Jo Moss, Lisa Nelson, Laura Groves, Chris Oliver

**Affiliations:** 1 Cerebra Centre of Neurodevelopmental Disorders, University of Birmingham, Birmingham, UK; 2 Institute of Cognitive Neuroscience, University College London, London, UK

**Keywords:** Cornelia de Lange, CdLS, Executive functioning, Behavioural phenotype

## Abstract

**Background:**

The aim of this study was to examine executive functioning in adolescents and adults with Cornelia de Lange syndrome (CdLS) to identify a syndrome and age-related profile of cognitive impairment.

**Methods:**

Participants were 24 individuals with CdLS aged 13–42 years (*M* = 22; SD = 8.98), and a comparable contrast group of 21 individuals with Down syndrome (DS) aged 15–33 years (*M* = 24; SD = *5.82*). Measures were selected to test verbal and visual fluency, inhibition, perseverance/flexibility, and working memory and comprised both questionnaire and performance tests.

**Results:**

Individuals with CdLS showed significantly greater impairment on tasks requiring flexibility and inhibition (rule switch) and on forwards span capacity. These impairments were also reported in the parent/carer-rated questionnaire measures. Backwards Digit Span was significantly negatively correlated with chronological age in CdLS, indicating increased deficits with age. This was not identified in individuals with DS.

**Conclusions:**

The relative deficits in executive functioning task performance are important in understanding the behavioural phenotype of CdLS. Prospective longitudinal follow-up is required to examine further the changes in executive functioning with age and if these map onto observed changes in behaviour in CdLS. Links with recent research indicating heightened responses to oxidative stress in CdLS may also be important.

## Background

Cornelia de Lange syndrome (CdLS) is caused by mutations of the NIP-BL gene on chromosome 5p13.1 for nearly 60% of individuals and by mutations on theSMC3, RAD21 and the X linked SMC1A and HDAC8 genes in a smaller proportion of affected individuals [1–5]. All five genes are involved in the structure and regulation of the cohesin complex which is crucial for neural maintenance and repair [2, 6]. It is probable that there are further unidentified mutations relevant to the cause of CdLS [7]. The physical phenotype of CdLS includes low birth weight, small stature, limb abnormalities, distinctive facial features, hearing and vision abnormalities, and cardiac, genito-urinary and gastro-intestinal disorders [8–10]. Degree of intellectual disability is variable and typically severe (30%) to profound (45%) with poor expressive relative to receptive language [11–14].

Behavioural research has focused on self-injurious behaviour and, more recently, autism spectrum disorder (ASD) characteristics (e.g. [13–16]). Reported prevalence rates of autism spectrum disorder in CdLS range from 43 to 67% [13, 14, 17–21]. However, the presentation of the triad of impairments in CdLS may not be typical of that observed in idiopathic ASD [19, 20]. Specifically, social impairment in CdLS is characterised by selective mutism, extreme shyness, social anxiety and social avoidance [11, 19, 22–24]. Although repetitive behaviours do not appear to contribute as significantly to the ASD profile, relative to social interaction and communication impairments [25, 26], they are characteristic of the syndrome, specifically lining up and tidying up behaviours and a strong preference for routine and need for sameness [15].

There is emerging evidence of age-related changes in behaviour in CdLS. Kline et al. [27, 28] reported 80% of individuals to show high levels of depression, self-injury, obsessive-compulsive behaviours, anxiety, aggression and hyperactivity. Blagowidow, Kline and Audette [29] also reported that behaviour disorder increased at puberty. Basile et al. [17] reported increased behavioural changes with age including communication disturbances and anxiety in 56 individuals aged 11 to 31 years, and Sarimski [12] found children over age six experienced significantly more social isolation and anxiety than younger children. Oliver et al. [25] reported that adults with CdLS were more likely to experience levels of negative affect and impulsivity (13%) compared to children with CdLS (3%), a profile not reported in other syndrome groups. Low mood is also reported within the teenage years compared to fragile X and Cri du Chat syndrome groups and is associated with higher scores on measures of insistence on sameness [30]. In combination, these observations of the behavioural phenotype of CdLS and its correlates suggest cognitive difference and change with age might be evident in this syndrome and related to the behavioural presentation.

Comparatively, less research has been published regarding the cognitive profile of CdLS. In this study, we will describe the cognitive profile, specifically executive function, in more able people with CdLS and consider the relationship with chronological age and its association with behavioural change. Executive functioning refers to cognitive abilities that control and regulate other abilities and behaviours and are necessary for goal-directed behaviour. They include the ability to initiate and stop actions, to monitor and change behaviour, and plan future behaviour when faced with novel tasks and situations. Executive functions allow individuals to anticipate outcomes and adapt to changing situations. The ability to form concepts and think abstractly are often considered components of executive function [31].

Specific deficits in executive functioning are thought to account for some of the observed behavioural problems seen in autism spectrum disorder [32] and are also described in a range of genetic syndromes such as fragile X (e.g. [32–36]), Prader-Willi, and Down syndromes. Woodcock, Oliver, and Humphreys [37] reported that impairment in attention switching in Prader-Willi syndrome was related to behavioural reports of adherence to routine and temper outbursts, providing evidence for an executive functioning-behaviour link. Similarly, changes in executive function that accompany decline in Down syndrome have also been described [38].

The frontal lobes are postulated to play a major role in executive functioning [39] and are the last part of the brain to fully develop. Studies of Prader-Willi, fragile X, and Williams syndromes reveal abnormalities in the frontal region that may be related to compromised executive functioning [35, 40]. For example, fMRI scanning of individuals with fragile X, in which difficulties with inhibition and visual attention switching are evident [35, 41], has shown reduced activation in the prefrontal cortex [42]. Brain imaging studies of individuals with CdLS are lacking. However, the few available autopsy studies have revealed frontal lobe hypoplasia in CdLS, indicating possible disorders of axonal growth, neural priming, and neuron cell repair [43]. Therefore, it may be postulated that impaired growth of the frontal lobes or emergent neuropathology may underpin behavioural change seen in CdLS, especially that occurring during adolescence and early adulthood [44].

Recent research by Gimigliano et al. [45] analysing the proteomic profile of the SMC1A and SMC3 genes implicated in CdLS showed protein expression was dysregulated. CdLS cell lines were found to show an increase in global oxidative stress as a result of this dysregulation, which the authors postulate could contribute towards the phenotype of the disorder, including premature aging and associated cognitive changes. They discuss how the subsequent reduced antioxidant defences could ultimately lead to cell death through DNA damage, membrane potential loss and reduced synthesis of ATP. These conclusions are tentative and require further investigation; however, the role of oxidative stress in neurodegeneration has been evidenced in other disorders. For example, oxidative stress in the brain has been associated with the pathogenesis of neuron degeneration and death in Alzheimer’s disease [46, 47]. It is therefore possible that oxidative stress may cause a chain of reactions that could account for the neuropathological and cognitive changes occurring with age in CdLS.

In summary, the available evidence suggests that behaviours that are phenotypic of a genetic syndrome may be underpinned by executive functioning impairments. The behavioural phenotype of CdLS also suggests a description of executive function deficits would be informative. Furthermore, reports of behavioural changes with age in CdLS, specifically around the age of adolescence/early adulthood, suggests that examining the association between executive function and age in CdLS would be informative as well.

A contrast group of individuals with Down syndrome (DS), comparable for age, gender, mobility, level of adaptive behaviour and receptive language (a domain not thought to tap into executive functioning [48]), was included in the current study. Down syndrome has a prevalence of 1 in 600 live births. The syndrome is caused by an extra 21st chromosome in 95% of people affected [49]. The physical phenotype includes epicanthic folds, protruding tongue, flat nasal bridge, brachcephaly, broad hands, brachydactyly and lax ligaments [50]. Developmental delay is also prevalent. At age 21, mean IQ is 42 (range 8–67) [50]. According to other studies (e.g. [51]), the behavioural and cognitive phenotype for DS includes relative strengths in elements of visuospatial processing [52, 53] and social functioning [54, 55], alongside relative deficits in language [56], verbal processing [57], verbal short-term memory and explicit long-term memory. However, visuospatial short-term memory, associative learning and implicit long-term memory functions are relatively preserved [58]. Executive functioning skills are considered to be broadly impaired adults with DS. Specifically, deficits in inhibitory control [59], flexibility [60] set shifting, sustained attention and planning have been documented in adults [61], alongside the aforementioned deficits in short-term memory. Fewer studies have evaluated these skills in children, although studies indicate that the profile of impairment may be similar to that observed in adults [62, 63]. According to Pritchard et al. [64], deficits in executive functioning in children and adolescents with DS are largely mediated by associated co-morbidities such as autism spectrum disorder symptomatology and ‘disruptive behavior disorder’. The role of intellectual disability in this association is not fully understood, and findings are inconsistent [62, 64].

In this study, a well-matched, homogenous group of people with DS, rather than a heterogeneous group of people with intellectual disability, was considered an appropriate contrast group. The literature describing cognition in individuals with DS is fairly consistent, and more is known about the behavioural and cognitive phenotype of DS than any other syndrome group. Therefore, this population provides a useful benchmark for profiling and interpreting the strengths and weaknesses in CdLS. This group can be positioned relative to the known areas of difficulty in individuals with DS. Due to the relatively small sample sizes, such a homogenous group was considered to add greater statistical power.

## Method

### Participants

Participants with CdLS were recruited either directly through a pre-existing research database of individuals with neurodevelopmental disorders who had previously participated in research and consented to be contacted in the future or through the CdLS Foundation (UK and Ireland). Participants with DS were recruited through the pre-existing research database only. Ethical approval was given by the University of Birmingham’s ethics committee.

Inclusion criteria comprised diagnosis from an appropriate professional (clinical geneticist or paediatrician), aged 12 years or over, able to speak at least 30 words, a self-help score indicating the person was at least partly able in self-help skills (indicated by scores on the Wessex Behaviour Scale [65] of seven or more out of nine), a receptive vocabulary age equivalent score of at least 40 months on the Vineland Adaptive Behavior Scale (VABS; [66]) and able to walk unaided. For practical reasons, participants were also required to live within 200 miles of the research base.

Thirty-four families of individuals with CdLS expressed an interest in participating in the study and were screened over the phone for eligibility. Five families did not participate due to distance, availability and illness, and one because they did not meet the inclusion criteria in relation to level of self-help skills. Of the 29 individuals visited, five had missing direct assessment data and a further two had missing questionnaire data. However, comparisons based on initial screening assessments indicated that these individuals did not differ from participants with full datasets on any of the inclusion criteria (*p* > .05). Twenty-four families of individuals with DS expressed an interest in participation. Of these, three families did not participate due to availability. In sum, 24 participants with CdLS (14 females and 10 males) aged 13–42 (*M* = 22; SD = 8.98) and 21 participants with DS (13 females and eight males) aged 15–33 (*M* = 24; SD = 5.82) years participated. Five participants with DS were missing VABS datasets due to failure to be able to contact the caregivers in the month following the visit; however, comparisons using Wessex scale inclusion criteria showed these individuals did not significantly differ to other participants on level of self-help skills (*p* > .05). Table 1 shows the demographic information of both groups. A comparison of the group demographics demonstrated that there were no significant group differences in relation to chronological age, gender, receptive language, adaptive behaviour skills and developmental quotient.

**Table 1:** A comparison of demographic information between the CdLS (*N* = 24) and DS (*N* = 21) groups

			CdLS	DS	t/χ^2^	*p*
Gender	% Female	% df	58.3 (1,45)	61.9	.06	.53
Age (years)		Mean (SD)	22.29 (8.98)	24.38(5.82)	−.91	.37
BPVS	Raw score		66.63 (20.23)	67.19 (23.70)	−.09	.93
	Age equivalence (years)		6.12 (2.15)	6.29 (2.72)	−.22	.83
Developmental quotient^a^			31.61 (12.59)	27.91 (10.29)	1.07	.29
VABS	Communication domain (standard score)		49.17 (16.74)	48.94 (24.36)	.04	.97
	Daily living skills (standard score)		55.96 (14.16)	56.0 (11.10)	−.01	.99
	Socialization (standard score)		56.87 (18.09)	52.56 (24.97)	.63	.53

^a^Calculated from receptive language age equivalences as measured by the BPVS (receptive language age/chronological age × 100)

### Measures

*Demographic Questionnaire*: A demographic questionnaire was used to obtain background information regarding age, gender and diagnostic status (i.e. whether and by whom the diagnosis was made by).

*The British Picture Vocabulary Scale—Second Edition* (*BPVS II;* [67]*)* was used to evaluate receptive vocabulary. The BPVS is reported to be psychometrically robust with good validity and reliability [67]. Age equivalence can be calculated. Developmental quotients for participants were calculated based on receptive language age equivalence and chronological age.

*The Vineland Adaptive Behavior Scale (VABS-II;* [66]*)* was used to assess adaptive behaviour (an individual’s personal and social skills as s/he interacts with her/his environment). The VABS is used widely for supporting the diagnosis of intellectual and developmental disabilities [66]. It is administered in a semi-structured interview format to the parents or caregivers. The test measures four main domains: ‘Communication’ , ‘Daily Living Skills’ , ‘Socialization’ and ‘Motor Skills’. The VABS was conducted face-to-face or via the telephone with the participant’s parent/carer.

### Measures of executive functioning

A range of individual tasks was used to assess different aspects of executive functioning abilities including working memory, verbal fluency, flexibility and inhibition skills. Additionally, a broad informant measure of executive functioning skills was completed by parents/carers. These assessments are outlined in detail below:

### Global measure of executive function

The *Behaviour Rating Inventory of Executive Function-Preschool Version (BRIEF-P;* [68]*)* is an informant-based questionnaire designed to examine deficits in several areas of executive function. The 63-item questionnaire is used to evaluate children with a wide spectrum of developmental and acquired neurological conditions. Given previous reports of low adaptive ability in individuals with CdLS and DS, the BRIEF-P was considered to be the most ecologically valid measure for the expected level of ability (similar to methodology used by Liogier d’Ardhuy et al., [69]). It is completed by a parent/carer who rates the person’s executive functioning within the context of everyday environments. It is reported to be an ecologically valid and efficient tool for screening, assessing and monitoring executive functioning. Ratings are made on a 3-point Likert scale (‘Never’ , ‘Sometimes’ , and ‘Always’) to indicate if a behaviour has been a problem over the last 6 months.

The five non-overlapping scales of the BRIEF-P are Inhibit, Shift, Emotional Control, Working Memory and Plan/Organize. These clinical scales form three indices: Inhibitory Self Control (Inhibition + Emotional control), Flexibility (Shift + Emotional Control) and Emergent Meta Cognition (Working memory + Plan/Organize) and one composite score (Global Executive Composite). The authors report high internal consistency (.80–.95) and test-retest reliability (.78–.90). *T* scores were calculated based on age equivalence scores of receptive language skills for each subscale and index. Where an individual’s receptive language ability was greater than the range assessed by the BRIEF-P, scores were normed on the highest age band available. Higher *T* scores indicate a greater deficit with scores of 65 or above indicating impairments that are of clinical significance.

### Assessments of working memory

Working memory consists of verbal and visuospatial subsystems [70]. Participants’ working memory capacity was examined using two tests designed to tap into each of these subsystems separately; the Digit Span test from the Wechsler Intelligence Scale for Children [71] and the Corsi Span test from the NEPSY [72].

*Digit Span from the Wechsler Intelligence Scale for Children—Third Edition UK (WISC-III;* [71]*).* The Digits Forward and Digits Backward tests comprise the Digits Span test, developed to measure working memory in typically developing children aged between 6 and 16 years. A participant’s score on the Digit Span task is the number of strings of digits they correctly recalled. The maximum length of the digit string correctly recalled was also recorded. The reliability and validity of this test has been reported to be good [71].

*Corsi Span: The Corsi Block-Tapping Test* (*From the NEPSY;* [72]*)* The Corsi Block-Tapping Task measures visuospatial short-term and working memory and is, arguably, the ‘single most important nonverbal task in neuropsychological research’ [73]. The Corsi blocks task was developed in the early 1970s as a visuospatial counterpart to the verbal-memory span task [74]. Participants watch the experimenter tap a sequence of blocks and then repeat the sequence themselves. In the backwards part of the task, they are to repeat the sequence in reverse order. Item one consists of two blocks to be tapped, and the number increases by one for each trial. A participant’s score on the Corsi Span task was determined by the number of correct trials. The maximum length of the string of block-taps they correctly recalled was also recorded.

### Assessments of fluency

Verbal and visual-spatial fluency were assessed using the Verbal Fluency test and the Design Fluency test from the NEPSY [72].

*Verbal Fluency from the NEPSY* [72]. This test assesses the ability to generate words quickly, according to semantic and phonemic categories. The Verbal Fluency test has been designed to assess fluency/generativity in typically developing children aged between 3 and 12 years old. The test is comprised of two parts: *Semantic Fluency* (listing as many words as possible in 60 s that are animals in trial 1 and food and drink in trial 2) and *Phonemic Fluency* (listing as many words in 60 s, excluding names of places and people, beginning with ‘S’ in trial 1 and ‘F’ in trial 2). The total raw score is calculated by summing up the number of correct words produced in each part of the test. The psychometric properties appear robust for this measure [75]. Scores on these Verbal Fluency tasks were determined by the number of novel words relating to a category that were generated in the 60-s time period. The number of repeated words is also examined to see whether there is more perseveration demonstrated within either of the groups.

*Design Fluency from the NEPSY* [72]*.* Design Fluency is a measure of nonverbal fluency. The test assesses ability to generate novel designs in a limited time period (60 s). It utilises executive functioning as participants need to plan and monitor their designs throughout the tasks, keeping the goal in mind. There are two tasks: participants are asked to connect two or more dots using straight lines to make a design on a structured array of dots, each contained in a separate box; and then do the same on an unstructured array of dots each contained in a separate box. Each design has to be different from the others.

The unstructured array increases executive load [75]. The total score is the number of novel designs generated. The number of repeated designs is also examined to see whether there is more perseveration demonstrated within either of the groups.

### Assessments of mental flexibility and inhibition

Flexibility and inhibition were assessed using the *Dimensional Card Change Sorting task (DCCS* [76]*)*. Participants are required to sort a series of bivalent cards, first according to one dimension (colour; red or blue), then according to the other (shape; boat or rabbit) and then according to whether or not a border is present. Three elements on the DCCS task produce three sets of scores; the number of correct card sorts (out of six) for colour and shape and then number of correct card sorts for shape/colour dependent on border (out of 12). Types of errors for the border task (e.g. colour and shape) were also recorded. No feedback was provided at any point.

The DCCS task is a widely used measure of executive functioning suitable for use with participants across a wide range of ages [77]. The majority of 3 years old successfully sort the cards on the first dimension, but demonstrate perseveration during the post-switch phase, exhibiting inflexibility [77]. By 5 years old, most children switch when instructed to do so. An additional challenge can be added for those participants who successfully switch to the new rule. They are given a ‘border’ version, whereby if a card has a border around it they are to sort by colour, if there is no border then they are to sort by shape.

### Procedure

Participants were assessed in their homes. On confirmation of the research visit, the participant’s parent/carer(s) were sent a questionnaire pack to complete. The first 20 min of the assessment session were spent building rapport and answering questions. Participants were given regular breaks throughout the testing. On completion of the test battery, participants and their families were given further opportunity to ask any questions, and debriefed.

### Data analysis

Data were checked to make sure they were normally distributed, and although there were some variables that were skewed (number of correct items in DCCS shape and border version), they followed the same pattern in both groups. To examine differences in the dependent variables, both MANOVA and ANOVA tests were used. Post hoc pair-wise comparisons were conducted where significant differences were identified. An exploratory analysis of the association between chronological age and executive function skills was conducted using the Pearson correlation.

## Results

### Executive functioning measures

#### Behaviour rating inventory of executive function

Table 2 shows mean *T* scores on each subscale and index of the BRIEF-P for each group. A one-way between groups MANOVA was performed to investigate syndrome group differences on BRIEF-P subscales and separately for BRIEF-P indices. There were no statistically significant group differences on the combined BRIEF-P subscale variables (*F*(5,34) = 1.21; *p* = .33; *η* ^*2*^ = .15) or the combined BRIEF-P indices variables (*F*(3,36) = 2.08; *p* = .12; *η* ^*2*^ = .15). When considered separately and using a Bonferroni adjusted alpha level of .01 for BRIEF-P subscales and .02 for BRIEF-P indices, there were no significant group differences identified for the BRIEF-P subscales. There was a significant between groups difference only in relation to the Flexibility Index (*F*(1,39) = 7.62; *p* = .01; *η* ^*2*^ = .16), with the CdLS group demonstrating significantly greater impairment in this domain compared to the DS group.

**Table 2:** Descriptives of the subscales and indices of the BRIEF-P

BRIEF-P Index		CdLS (n = 22)	DS (n = 18)
Subscales			
Inhibition	Mean (SD)	56.00 (10.51)	51.58 (8.57)
Shift		64.77 (10.75)	56.95 (10.67)
Emotional Control		58.27 (11.12)	50.26 (11.19)
Working Memory		64.27 (13.37)	58.78 (11.99)
Plan/Organise		57.09 (10.28)	53.37 (10.98)
Indices			
Inhibitory self control	Mean (SD)	57.68 (11.18)	51.11 (8.14)
Flexibility		63.14 (10.27)	54.16 (10.52)
Emergent Meta cognition		62.23 (12.38)	57.28 (11.70)
Global Executive composite		62.68 (11.16)	55.83 (9.37)

Scores of ≥65 suggests impairments are of clinical significance

For both groups, no subscale score met criteria for clinical significance (scores of ≥65); however, the CdLS group’s Shift and Working Memory subscales did approach the cut off. The CdLS participants’ scores also approached clinical cut off for the Flexibility and Emergent Meta cognition indices and for the Global Executive composite.

#### Working memory

Table 3 shows the results from the Digit Span, Corsi Span, Verbal Fluency and Design Fluency tests.

**Table 3:** Results of the Digit Span, Corsi Span and Fluency subtests

			CdLS (n = 24)	DS (n = 21)
Digit Span test				
Forward	Number correct	Mean (SD)	5.56 (2.81)	6.67 (2.20)
	Maximum length		3.11 (1.02)	3.67 (0.80)
	Number of individuals scoring ≤2		5	0
Backward	Number correct		2.47 (1.91)	2.52 (2.16)
	Maximum length		1.94 (1.25)	1.62 (1.36)
	Number of individuals scoring ≤2		11	17
Corsi Span test				
Forward	Number correct	Mean (SD)	4.52 (3.27)	7.05 (3.29)
	Maximum length		2.90 (1.51)	4.00 (1.26)
	Number of individuals scoring ≤2		8	3
Backward	Number correct		3.53 (3.20)	3.16 (2.04)
	Maximum length		2.33 (1.85)	2.29 (1.35)
	Number of individuals scoring ≤2		12	13
Verbal Fluency				
Animals	Number generated	Mean (SD)	7.35 (6.03)	8.14 (4.67)
Food & Drink	Number generated		6.85 (6.00)	10.86 (5.02)
Design Fluency				
Structured Array	Correct	Mean (SD)	4.70 (3.18)	6.10 (3.03)
Random Array	Correct		5.09 (3.50)	6.19 (3.48)

Three factor mixed ANOVAs with group (DS, CdLS) as the between subject factor and domain (verbal, spatial) and direction of recall (forward, backward) as within factors were carried out to evaluate differences in maximum span length and separately for number of correct trials. Pair-wise post hoc comparisons were conducted where main effects and interactions were significant. In relation to maximum span length, analysis revealed a significant main effect of domain (*F*(1,36) = 10.68; *p* = .002; *η* ^*2*^ = .23), with significantly longer spatial spans compared to verbal spans in both syndrome groups (*p* < .001). There was also a significant main effect of recall direction (*F*(1,36) = 70.19; *p* < .001; *η* ^*2*^ = .66), with all participants having significantly longer forward spans compared to backward spans (*p* < .001), and a significant group by recall direction interaction (*F*(1,36) = 11.59; *p* = .002; *η* ^*2*^ = .24). Post hoc analyses revealed that individuals with CdLS had significantly shorter forward spans compared to individuals with DS (*p* = .03) while no significant differences were identified on the backward span tasks. A marginally significant recall direction by domain interaction was identified (*F*(1,36) = 4.99; *p* = .03; *η* ^*2*^ = .12), with spatial backward spans significantly longer than verbal backward spans while no significant differences between spatial and verbal forward spans was identified. There were no other significant main effects of interactions in relation to length of digit span. These results suggest that for both groups spatial digit span is better than verbal digit span and that forward span is generally better than backward span. However, individuals with CdLS are comparatively more impaired than individuals with DS in relation to forward span while both groups are similarly impaired in relation to backward span. In both groups, scores in relation to backward span were close to floor.

Results from the analysis conducted for number of correct trials were the same as those revealed for span length. There was significant main effect of recall direction (*F*(1,36) = 111.41; *p* < .001; *η* ^*2*^ = .76), with significantly more correct trials for forward span relative to backward span across all participants (*p* < .001). The interactions between recall direction and group and between recall direction and domain were significant (*F*(1,36) = 11.44; *p* = .002; *η* ^*2*^ = .24; *F*(1,36) = 7.18; *p* = .01; *η* ^*2*^ = .17).

#### Fluency

Scores on the phonemic fluency tasks were very low with both groups generating only two to three words in each category within the 60 s. These data were therefore not included in the analysis. Verbal fluency was evaluated by recording the number of words generated in 60 s. Repeated measure ANOVAs were conducted in order to evaluate whether it was possible to collapse the two semantic trials (animals and food) into a single semantic category. A significant group by condition interaction (*F*(1,39) = 7.54; *p* = .01; *η* ^*2*^ = .16) was identified for semantic fluency, indicating that group had a differential effect on number of words generated for animal and food/drink trials, and trials were therefore examined separately. *T* tests revealed that the CdLS group generated fewer words on the food/drink fluency trials than the DS group. This difference was marginally significant (*t*(1,39) = −2.32; *p* = ..03; *η* ^*2*^ = .12).

A mixed ANOVA with condition (structured, unstructured) as the within subjects factor and group (CdLS, DS) as the between subjects factor was conducted to evaluate nonverbal fluency skills. Analysis revealed no significant main effects of condition (*F*(1,42) = .76; *p* = .39; *η* ^*2*^ = .02) or group (*F*(1,42) = 1.71; *p* = .20; *η* ^*2*^ = .04) and no condition by group interaction (*F*(1,42) = .28; *p* = .60; *η* ^*2*^ = .01).

In relation to planning and self-monitoring on the design fluency task, both groups showed deficits. The CdLS group had a mean of 3.26 (*SD* = 5.22) designs that were repeats of the other designs in the structured array, and the DS group had 3.76 (*SD* = 5.05), over half of the number of correct designs. The same was true for the random array; CdLS (*M* = 3.44, *SD* = 4.05) and DS (*M* = 4.52, *SD* = 5.06). This suggests both individuals with CdLS and DS have difficulty planning and monitoring their goals.

#### Dimensional change card sort

**Fig. 1:**
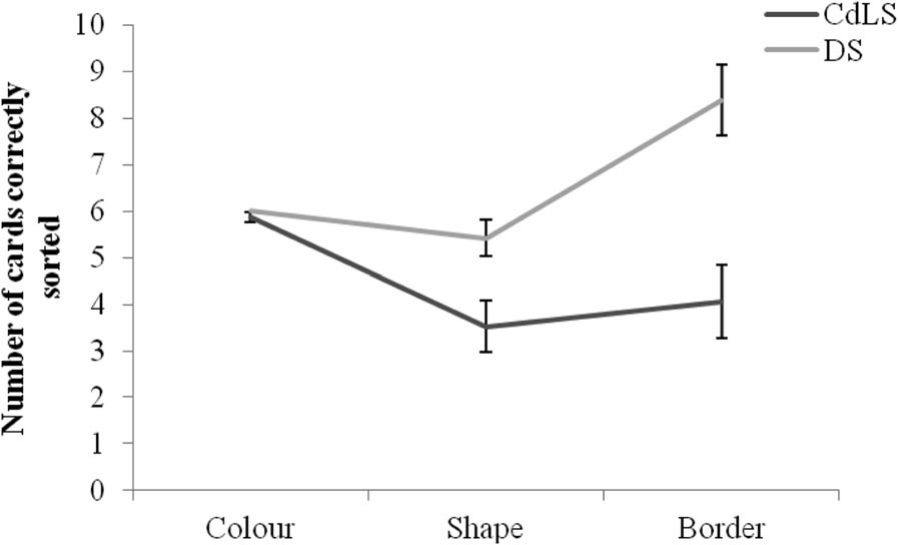
Number of cards correctly sorted for the colour, shape and border elements of the DCCS task. Total number of cards in each element (*colour*, *shape and border*) was 6, 6 and 12 respectively. Only individuals who successfully switched from the colour to the shape rule progressed to the border version (CdLS *n* = 13, DS *n* = 19)

The results of the DCCS are shown in Fig. 1. A mixed ANOVA with sort parameter (colour, shape, border) as the within group factor and syndrome group as the between group factor revealed a significant group by parameter interaction (*F*(2,40) = 8.06; *p* = .001; *η* ^*2*^ = .29) and significant main effects of group (*F*(1,41) = 13.21; *p* = .001; *η* ^*2*^ = .24) and parameter (*F*(2,40) = 26.61; *p* < .001; *η* ^*2*^ = .57). Post hoc pair-wise comparisons showed that both the CdLS and DS groups were able to correctly sort the six cards according to colour in the first part of the task (CdLS *M* = 5.86, *SD = .47*; DS *M* = 6.00, *SD* = .00; *p* = .19). When the rule was changed to sort for shape, the participants with CdLS sorted significantly fewer cards according to this rule (*M* = 3.52, *SD* = 2.63; *p* = .01), compared to the DS group (*M* = 5.43, *SD* = 1.81), continuing to sort by colour for some of the cards. The final part of the task required participants to sort by colour if the picture was surrounded by a border, and shape if there was no border. Only participants who had correctly sorted two out of the six cards in the previous element of the task (shape) participated in this final element (*n* = 19 for DS; *n* = 13 for CdLS). Again, the DS group (*M* = 8.38, *SD* = 3.46) performed significantly better than the CdLS group (*M* = 4.05, *SD* = 3.70; *p* < .001), suggesting that individuals with CdLS may have difficulty in flexibility and inhibition.

Closer analysis of the data revealed a significant between groups difference on the border element of the task with regard to the nature of the errors being made. The CdLS group were mainly making the error of continuing to sort shape cards by colour (*M* = 2.46, *SD* = 1.51; *F*(1, 30) = 7.64, *p* = .01, *η* ^*2*^ = .20), the original rule, providing evidence of perseveration in the CdLS group.

#### Association between age and executive functioning

Correlations between chronological age (CA) and measures of executive functioning were examined as part of an exploratory analysis to evaluate risk for changes in executive function with age. For the CdLS group, CA was negatively correlated with maximum span on the Digit Span Backwards task (*r* (17) = −.50, *p* = .04). This suggests that older participants with CdLS may have poorer verbal working memory than younger participants with CdLS.

Interestingly, the DS group showed a significant positive correlation between CA and the number of correct items on the border element of the DCCS (*r* (21) = 51, *p* = .02) and a significant negative correlation between CA and the number of items on the task that were incorrectly sorted i.e. according to the original rule (*r* (19) = −.59, *p* < .01). This suggests that as people with DS get older their rule switching abilities improve. No other correlations were significant.

## Discussion

This study details the results of the first evaluation of the executive functioning profile in more able adolescents and adults with CdLS. Participants with CdLS were compared to participants with DS, using several measures of executive functioning. Initial comparisons indicated that the two participant groups did not differ with regard to gender, chronological age, adaptive behaviour and receptive language age equivalence scores.

Carer-rated BRIEF-P *T* scores revealed that participants with CdLS demonstrated a similar profile of executive functioning to the DS group but with significantly greater impairments in flexibility. This suggests that individuals with CdLS are broadly compromised in executive functioning skills, to a similar degree as has been previously demonstrated in those with DS [78]. On some subscales, scores in the CdLS group approached clinical significance (e.g. Shift and Working Memory). As raw scores were normed on a group of typically developing children who were younger than reported age equivalence scores of some of the CdLS and DS participants, the magnitude of executive functioning deficits may have been weakened. This could explain why clinical significance was not reached. Currently, there is no informant report assessment of executive functioning which considers both a person’s chronological age and their developmental age, which is something future studies should address. The fact that individuals with CdLS are significantly more impaired than the matched DS sample in the Flexibility Index is indicative of a specific syndrome group difference. Direct assessment, using the DCCS, confirmed that participants with CdLS demonstrated significantly greater difficulty in flexible thinking compared to the DS group, continuing to sort cards by the first rule of the task, rather than shifting to the new rules as the assessment progressed. Given that there were no significant group differences in phonological or spatial working memory (as measured by the backwards Digit Span and Corsi Span tests, respectively), the difficulties in rule shifting evident in the CdLS group are unlikely to be related to learning and retaining the rule of the task but in perseveration and inflexibility. Equally, it is unlikely that these differences are attributable to degree of disability, as both groups were well matched on receptive language, adaptive behaviour skills and developmental quotient. In relation to working memory skills, both groups were limited in their span capacity expected given their chronological age (both having spans of three). In typically developing populations, working memory capacity typically increase from a span of three at 4 to 5 years of age to a span of seven to eight at 16 years [79]. Impairments in capacity of the phonological loop impact on speech and language development [80], which is consistent with the developmental profile of these two syndrome groups. Further analyses suggested that both groups were more impaired on verbal working memory relative to spatial working memory; however, without a typically developing sample, it is difficult to determine whether this is evidence of atypical working memory abilities in these syndromes. Individuals with CdLS were significantly more impaired than individuals with DS on the forward span tasks (across both domains) while both groups were similarly impaired (and in fact scoring almost at floor level) on the backward span tasks. For the DS group, the increase in the number of individuals scoring at floor from the forwards to the backwards span tasks further demonstrates difficulties in working memory across both domains. A similar effect was observed in the CdLS group; however, there were notably more individuals scoring at floor level on the forward span tasks than the DS group. This may suggest individuals had difficulties understanding task demands; however, the decrease in scores when switching from the forwards to the backwards tasks is consistent with the increase in difficulty which suggests the low scores are an accurate reflection of the individuals’ difficulties in task performance. Verbal fluency was marginally poorer in the CdLS group compared to the DS group, while both groups had difficulty with the more complex fluency tasks which required a greater capacity for organising concepts in a novel way [34]. The differences in verbal fluency are particularly interesting given that there were no significant group differences on the receptive language skills that might confound performance on such tasks. Results from the design fluency task revealed little difference between the two groups. Both groups appeared to show difficulties in monitoring their drawings, with many repeated designs being drawn. This suggests that difficulties in planning and monitoring are characteristic of both syndrome groups and this might be accounted for by global cognitive impairments rather than a syndrome-related executive function deficit. However, further investigation with a matched typically developing contrast group would be needed to fully determine the nature of this deficit in both groups.

Overall, there is large overlap in the executive functioning profiles of individuals with CdLS and DS. However, there are also marked group differences on very specific domains of executive functioning which may represent syndrome specific deficits in CdLS. The profile of differences in executive functioning in individuals with CdLS is consistent with higher prevalence of repetitive behaviours, specifically tidying up and lining up behaviours in individuals with CdLS and the strong insistence on routine and need for sameness described by Moss et al. [15]. According to Turner [81], these behaviours may all be explained by deficits in executive functioning skills. This theory is supported by findings that deficits in executive function are observed in a range of other neurodevelopmental disorders with increased repetitive behaviour including autism spectrum disorder, Prader-Willi syndrome and Fragile X syndrome [32, 37, 82].

Some of the CdLS participants failed to engage in all of the tasks. Those who did not complete the tasks were not significantly different to those that did, with regards to the characteristics described in Table 1 (*p* > .05 for all variables). Since there were no overall group differences in verbal working memory, it is unlikely that failure to complete tasks was due to weaknesses in recalling instructions. It is tentatively suggested that problems with task initiation may account for the failure of some participants to engage in the tasks. This is supported by the authors’ clinical observations and by anecdotal research observations describing difficulties in task initiation [83]. It needs to be determined whether this is due to social/performance anxiety that is also characteristics of this group [84] or whether this is determined by the specific executive functioning difficulties that appear to be characteristics of this group. However, it is not clear why these difficulties in task engagement were not consistent across all executive functioning tasks.

A significant correlation between age and backwards digit span suggests that older individuals with CdLS have poorer verbal working memory compared to younger individuals with CdLS; although, caution should be taken when interpreting this finding given the exploratory nature of the analysis. This pattern of results is consistent with previous research which has also highlighted changes in mood, behaviour and cognition with age [26, 83, 85] and reported physical signs of premature ageing [28]. The compromised function of the cohesin pathway (resultant from the genetic mutations which cause CdLS) has been implicated in these behavioural and cognitive changes in CdLS, due to the role of this pathway in neural maintenance and repair [28]. Recent evidence also indicates downregulation of proteins involved in the response to oxidative stress and an increase in global oxidative stress in CdLS cell lines which may be directly linked to the phenotypic changes in the syndrome [86]. A larger sample with a wider age range and prospective longitudinal follow-up is needed to examine the association between age and executive functioning in more depth as it may be that individuals in the sample were already experiencing some age-related changes so correlations may have been weaker as a result. It is of course also worth considering ascertainment effects in relation to age-related findings. Greater understanding about the condition and better diagnostic testing may lead to earlier diagnosis, and identification of milder forms of CdLS than was previously the case, so it may be argued that any differences related to age could be attributed to the older groups having more severe difficulties. More research with larger samples is clearly indicated.

The findings should be considered within the context of some methodological shortcomings. Primarily, the use of a group of participants with DS warrants comment. One difficulty with using this group is their increased risk of Alzheimer-related dementia. Alzheimer-related dementia in DS has a prevalence of 0–2% in individuals under 40 years old and more than 40% in those over 60 years [58]. The later stages of dementia in DS are well documented in the literature while research into the earlier and likely more subtle cognitive and behavioural changes that may occur in the initial stages of dementia in DS has only started to emerge in recent years (see [38]). Although the DS sample in the current study were all under the age of 40, thus mitigating the likelihood of dementia, it remains a possibility that some of these individuals may have been affected by the early stages of dementia which could impact on their performance. Independently of the association with Alzheimer-related dementia, research studies suggest that both children and adults demonstrate broad impairments in executive functioning skills [60–63, 87]. Therefore, where no significant group differences were identified in the current study, we can determine that individuals with CdLS demonstrate a similar degree of impairment in these skills relative to those with DS; however, we cannot determine fully the extent of this impairment and how it is related to the presence of an intellectual disability. Future research should include a typically developing contrast group that would enable the degree of impairment in CdLS to be determined more precisely. However, with both of these limitations in mind, it is poignant that the CdLS group performed significantly worse than the DS group on specific aspects of executive function and noteworthy that the two groups demonstrated equal abilities in these areas.

The sample size of each group was relatively small which may have comprised statistical power. Also, whilst a variety of tests were used to examine different aspects of executive functioning, there are some elements of executive functioning that have not been addressed in the current study and as such will need to be looked at in future research to complete the picture of executive functioning in these groups. There was also no measure of motor abilities in the current study. This would be a useful measure to use in future research so as to rule out difficulties in motor skills as explaining difficulties with tasks requiring the participants to draw or write, for example Design Fluency.

## Conclusions

This study has addressed questions about the domain asynchrony and syndrome-related executive functioning in CdLS and DS. Three dimensions of executive functioning, flexibility/task-switching, inhibition and fluency, were found to be significantly impaired in individuals with CdLS relative to a matched DS contrast group, indicating that these are potential areas of syndrome-related deficit. With regard to domain asynchrony, it is not yet clear how the impairments in elements of executive functioning in CdLS combine to affect day-to-day behaviours. Current evidence points to these deficits as a contributing to repetitive behaviours evidenced within the syndrome. However, this warrants further investigation. The study also highlighted that deficits in executive function abilities may become more prominent with chronological age, providing support for previous studies describing behaviour, cognitive and physical change in this group. More research with larger sample sizes is clearly indicated to examine this further, and to rule out potential ascertainment effects.

From a pedagogical perspective, this research can begin to inform the design of more effective education and rehabilitation strategies that are tailored to the syndrome [59]. For example, there may be strategies to help develop executive functioning in different areas or to help compensate for deficits, so optimising a person’s potential.
